# Congenital neck cyst originated from ultimobranchial body in a fetus^☆^

**DOI:** 10.1016/j.bjorl.2016.03.005

**Published:** 2016-04-22

**Authors:** Jong Seung Kim, Jong Seok Oh, Yong Joo Yoon, Min Hee Lee, Eun Jung Lee

**Affiliations:** a Research Institute of Clinical Medicine of Chonbuk National University, Biomedical Research Institute of Chonbuk National University Hospital, Department of Otolaryngology-Head and Neck Surgery, Jeonju, Republic of Korea; b Presbyterian Medical Center, Department of Internal Medicine, Division of Allergy and Pulmonology, Jeonju, Republic of Korea

## Introduction

Using intrauterine ultrasonography here is increasing evidence of detection of structural abnormalities that influence prenatal treatment and prognosis.

The role of prenatal ultrasonography in the fetal neck mass is to observe the size and position change of the mass through continous echogenicity and to plan the treatment protocol of delivery timing and method by a thorough inspection of the adjacent trachea, esophagus and larger vessels.

We report a huge thyroid cyst in a fetus diagnosed by prenatal ultrasonography at 31st pregnant week. He suffered from postnatal apnea and a feeding disturbance which an operation on the 15th day after birth. Histopathology showed a cystic mass with a capsule that is composed of squamous epithelial cells, which is adherent to the postero-superior portion of thyroid. Herein we report the first known case of a thyroid cyst originated from the ultimobranchial body.

## Case report

A 35-year-old second gravida female at 31 weeks 6 days of gestation was transferred to our institution presenting a 3.6 cm × 1.8 cm fetal cystic neck mass diagnosed by fetal ultrasonography at an obstetric clinic. She had a history of endometriosis in 2001 and delivered her first baby through cesarean section in March 2003. The patient was not on any medication. Systemic examination was normal. Ultrasound examination revealed a 4.54 cm × 1.39 cm cyst of the fetal neck with normal amniotic fluid and normal fetal development. The mass was found behind the left trachea, showing no difference in size and shape on two occasions of follow-up ultrasonography, each done three weeks apart. At thirty-nine weeks of gestation, the patient was posted for cesarean section, and delivered a healthy male baby of 3240 g (50–75 percentile). The APGAR score was 9 point at 1 min and 10 points at 5 min. There were no gross external deformities or anomalies in the neonate.

**Figure 1:**
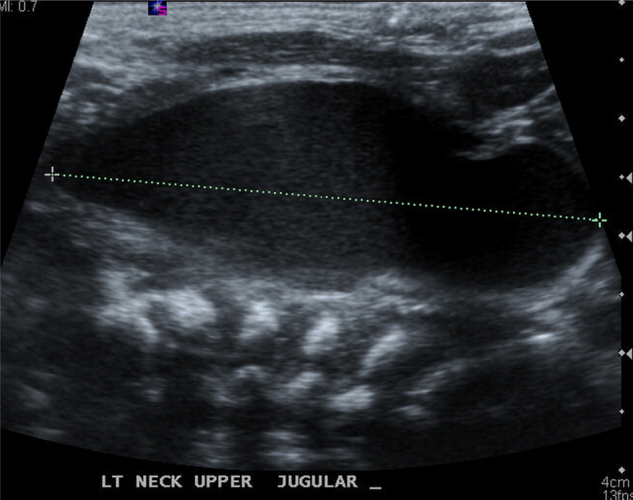
On ultrasound scan the neonatal huge neck mass (4.0 cm × 2.3 cm × 1.5 cm) was described as cystic mass with anechoic area.

**Figure 2:**
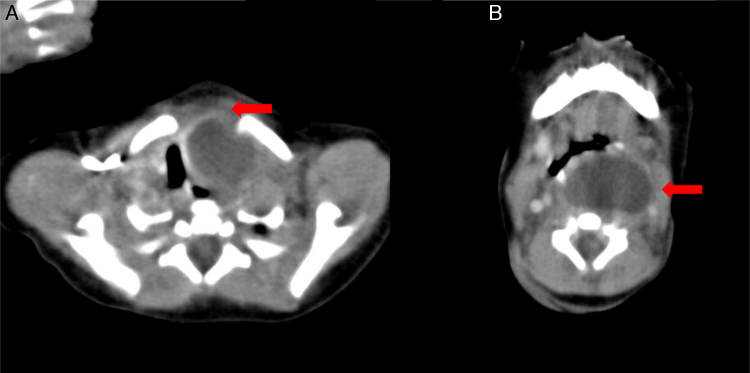
Preoperative computed tomography scan shows 4.5 cm × 3 cm size unilocular low density cystic mass which is extended into superior mediastinum with compressing the trachea. A, red arrow: intrathoracic lesion. B, red arrow: superior lesion.

We suspected a tracheo-esophageal fistula. An esophagogram using gastrograffin was performed at two days after birth, which showed no evidence of fistula and normal esophageal passage. On the 3rd day after birth, the infant showed decreased feeding with crying. On the 4th day, several episodes of intermittent airway obstruction without cyanosis were observed. Arterial blood gas analysis was normal and the infant was fed by nasogastric tube. On the same day, a 2.3 cm × 1.3 cm cystic mass was observed in a neck ultrasonogram, which was positioned lateral to the left thyroid and protruding to the left carotid artery and internal jugular vein (Fig. 1). The initial differential diagnosis was changed to a thymic cyst, teratoma or 4th branchial cleft cyst and the patient was referred to our department on the 7th day after birth. Computed tomography (CT) revealed a unilocular cystic mass which occupied the retropharyngeal space superiorly extending the thoracic inlet inferiorly (Fig. 2). Pre-operative differential diagnosis was thyroid cyst or thymic cyst and the operation was planned under general anesthesia on day 15.

**Figure 3:**
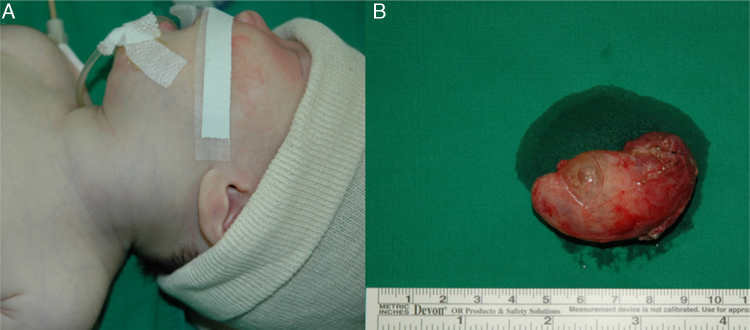
Patient photograph of the operating field. Note the diffuse swelling of the left anterolateral neck (A). About 5 cm × 2 cm × 2 cm size soft, lobulated cystic mass which was filled with clear yellowish colored fluid (B).

**Figure 4:**
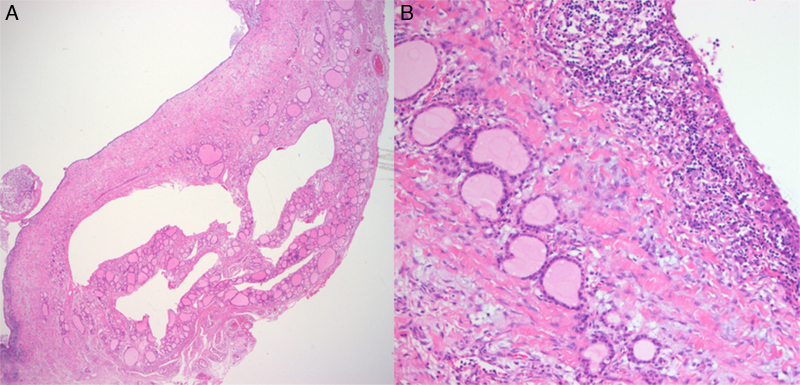
Histological finding of ultimobranchial body cyst in thyroid gland (A, 100×). The cyst is lined by stratified squamous epithelium that is slightly larger than thyroid follicular cell with surrounding small lymphocytes (B, 400×).

During examination in the operation room, there was found a soft mass with a diffuse swelling on the left anterior neck (Fig. 3). After a transverse cervical incision, a yellow cystic mass with a thin capsule that had no tract or fistula superiorly was exposed. Partial thyroidectomy was done due to the adhesion to the posterosuperior part of left thyroid. The recurrent laryngeal nerve, phrenic nerve and internal carotid artery were identified and preserved in the operating field. The dimension of the mass was 4.5 cm × 2 cm × 2 cm and there was white greenish colored cystic fluid inside the mass. Histopathology confirmed a huge thyroid cyst with a lining of squamous epithelium (Fig. 4).

## Discussion

A congenital lateral neck mass can originate from the obliteration failure of embryologic remnant. The second type of branchial cleft anomaly is the most common one. In 1983, Burge and Middleton proposed to term it as “a pyriform sinus cyst”, in that most congenital lateral neck mass is the fourth branchial cleft cysts.^1^ However, congenital lateral neck masses originating from the ultimobranchial body remnant is very rare.

Ultimobranchial body (UBB) stems from the 4th and 5th branchial pouch and is the origin of parafollicular C cell. Parafollicular C cell produces calcitonin in the thyroid and lowers the calcium level of serum.^2^ UBB integrates into the thyroid when the thyroid differentiates into two lobes to ultimately position at the location of thyroid cartilage at 10th to 12th embryonic week. At times, it also remains a cystic structure until the end of the fetus period.^3^ The position of UBB is studied by calcitonin positive cells in the thyroid if the mass has no embryonic correlation with no trace of UBB. McMillian et al. analyzed calcitonin containing cells in adult thyroids by immunostaining and confirmed that there were conglomerated areas of calcitonin- containing cells in the center of lateral thyroid lobe.^4^ Wolfe et al. identified the increased expression in the upper half of both thyroid lobe by radioimmunoassay.^5^ Fraser and Duckworth revealed the position of UBB is upper half of both thyroid lobes near the upper parathyroid.^6^ He knew the UBB exists in the form of cyst and conducted an autopsy of 21 fetus thyroids through 7 μm thickness serial section. Roediger et al. first reported that the position of the congenital thyroid cyst is postero-superior portion of thyroid.^7^ However, he also identified a connection with oropharynx, which cannot rule out the fourth branchial cleft cyst. This case is a pure cyst that has no tract or fistula formation superiorly; the adhesion to the postero-superior portion of thyroid implies the origin is UBB.

Further evidence this cyst is a UBB remnant is the histopathology. The incidence of UBB remnant is 87.5% (Sugiyama et al.)^8^ who studied 96 thyroid lobes of fetal autopsy, 60% by Harach^9^ and 89% by Beckner et al.,^10^ who studied 18 fetal autopsies. UBB remnant is known as solid cell nest due to its morphologic characteristic,^9^ however, many authors reported the cystic structure of UBB remnant.^6^, ^8^, ^10^ In this regard, solid nest cell is a misnomer. Histologically, classic UBB remnant forms a small solid nest comprised of larger cell than the adjacent follicular cells. Many authors reported that the cell component is squamous cell, Frazer^6^ and Roediger^7^ pointed out that the epithelium of the cyst is squamous epithelium and occasionally stratified squamous or ciliated cuboidal epithelium. We suspected the origin of this tumor is UBB remnant, because the histology showed small lymphoid cells forming a true cyst and the lining cell was stratified squamous epithelium.

It is rare that a cystic thyroid lesion occurs the pediatric age group and very exceptional that it is detected as lateral neck mass by prenatal ultrasonography. This is due to universal ultrasonography, allowing for the regular observation of fetal development and screening of the structural abnormality. Fetal neck masses can cause many problems like asphyxia after birth, dystocia and hemorrhage due to hydroamniosis. Prenatal ultrasonography is essential to evaluate neck masses and to plan the delivery timing and approach.

## Conclusion

This report is meaningful in that the congenital neck cyst was early detected by early perinatal ultrasonography. Histologically, the origin of the cyst is the UBB remnant, which is rarely reported.

## Conflicts of interest

The authors declare no conflicts of interest.

## References

[1] Burge D., Middleton A. (1983). Persistent pharyngeal pouch derivatives in the neonate. J Pediatr Surg.

[2] Chan A.S., Conen P.E. (1971). Ultrastructural observations on cytodifferentiation of parafollicular cells in the human fetal thyroid. Lab Invest.

[3] Sugiyama S. (1969). Embryonic development of human thyroid gland and ultimobranchial body. Acta Endocrinol (Cph).

[4] McMillian P.J., Hooker W.M., Deftos L.J. (1974). Distribution of calcitonin containing cells in the human thyroid. Am J Anat.

[5] Wolfe H.J., Melbin K., Cervi-Skinner S., Al Saadi A.A., Juliar J.F., Jachson C.E. (1973). C-cell hyperplasia preceding medullary thyroid carcinoma. N Engl J Med.

[6] Fraser B.A., Duckworth J.W. (1979). Position of ultimobranchial body cysts in the human fetal thyroid gland. Acta Anat.

[7] Roediger W.E., Kalk F., Spitz L., Schmaman A. (1977). Congenital thyroid cyst of ultimobranchial gland origin. J Pediatr Surg.

[8] Sugiyama S. (1971). The embryology of the human thyroid gland including ultimobranchial body and others related. Ergeb Anat Entwicklungsgesch.

[9] Harach H.R. (1985). Solid cell nests of the thyroid. An anatomical survey and immunohistochemical study for the presence of thyroglobulin. Acta Anat.

[10] Beckner M.E., Shultz J.J., Richardson T. (1990). Solid and cystic ultimobranchial body remnants in the thyroid. Arch Pathol Lab Med.

